# MicroRNA-34c-5p exhibits anticancer properties in gastric cancer by targeting MAP2K1 to inhibit cell proliferation, migration, and invasion

**DOI:** 10.1155/2022/7375661

**Published:** 2022-09-27

**Authors:** Qian Ma, Yuan Zhao, Zhaojun Li, Wenwei Gao, Yuanyi Xu, Bing Li, Yunning Huang, Zhenghao Huo

**Affiliations:** ^1^School of Basic Medicine, Ningxia Medical University, Yinchuan750000, China; ^2^College of Life Sciences, Ningxia University, Yinchuan750000, China; ^3^School of Nursing, Ningxia Medical University, Yinchuan750000, China; ^4^Department of Gastrointestinal Surgery, The Affiliated People's Hospital of Ningxia Medical University, Yinchuan750000, China

## Abstract

**Purpose:**

Gastric cancer(GC)is one of the deadliest digestive tract tumors worldwide，existing studies suggest that dysregulated expression of microRNAs (miRNAs) plays an important role in the pathogenesis and progression of GC. This study aimed to investigate the expression, biological function, and downstream mechanism of miR-34c-5p in GC, provide new targets for gastric cancer diagnosis and treatment.

**Methods:**

The expression of miR-34c-5p in GC tissues and cell lines was examined by RT-qPCR. Cell wound healing, transwell and cell cloning assays were used to detect the effect of miR-34c-5p on the migration and invasion abilities, respectively, of GC cells. Western blot was performed to detect the expression of related proteins. Bioinformatics analysis was used to predict the binding of MAP2K1 to miR-34c-5p and the targeting relationship was confirmed by dual luciferase reporter assay.

**Results:**

The expression level of miR-34c-5p was significantly decreased in GC tissues and cell lines. miR-34c-5p overexpression inhibited migration, invasion, and colony formation of gastric cancer cells, the related protein E-cadherin expression was significantly increased and N-cadherin, vimentin, and PCNA expression were significantly decreased, while miR-34c-5p knockdown exerted the opposite effects. In addition, the targeting relationship between miR-34c-5p and MAP2K1 was predicted and confirmed, and further confirmed by rescue experiments that MAP2K1 alleviated the inhibitory effect of miR-34c-5p in GC.

**Conclusion:**

MiR-34c-5p is lowly expressed in GC, and it can target MAP2K1 to exert inhibitory effects on GC proliferation, invasion, and migration. These findings provide a promising biomarker and a potential therapeutic target for gastric cancer.

## 1. Introduction

Gastric cancer (gastric cancer，GC) is one of the most common gastrointestinal tumors [1, 2], which has a high mortality rate and a low 5-year survival rate due to its low early diagnosis rate and susceptibility to abdominal implantation and metastasis [3, 4]. Therefore, investigating the molecular mechanism of gastric carcinogenesis and finding effective diagnostic and therapeutic targets are of vital importance for the early diagnosis of GC and improving patient prognosis.

MicroRNAs (miRNAs) are a group of small single-stranded RNAs about 18-25 nucleotides，which regulate protein expression at the posttranscriptional level through reduction of mRNA stability or inhibition of translation via binding to the 3'-untranslated regions (3'UTRs) of target genes, and miRNAs are involved in regulating the biological behavior of a variety of tumors, including GC [5–8]. For example, miR-29c inhibits the proliferation of GC cells by targeting NASP [9], miR-370 inhibits the invasion and migration of hepatocellular carcinoma by targeting regulatory GUCD1 [10].

In the present study, we supposed that the dysregulated miR-34c-5p had an inhibitory effect on migration, invasion, and colony formation of GC cells. Furthermore, miR-34c-5p was unveiled to have a binding site on MAP2K1 3'UTR. Therefore, we hypothesized that miR-34c-5p may play a critical role in GC progression by targeting and regulating MAP2K1. Thus, the study aims to elucidate the function of miR-34c-5p on gastric cancer progression and the underlying mechanism. Our results provide new insights into miRNAs in GC and offer a theoretical basis for the clinical treatment of GC.

## 2. Materials and Methods

### 2.1. Clinical samples

Fifty human GC tissue samples and paired normal tissues were obtained from GC patients who received radical gastrectomy in People's Hospital of Ningxia Hui Autonomous Region from July 2019 to July 2021. The patients with no history of other tumors and no other adjuvant treatment before surgery and the samples were stored in liquid nitrogen immediately after surgical resection for further analysis. This study was approved by the Ethics Committee of Ningxia Medical University, and all patients participating in the current study gave informed consent.

### 2.2. Cell culture

Human normal gastric mucosa epithelial cell line GES-1 and poorly differentiation gastric cancer cell line MKN-45 were purchased from Shanghai Zhong Qiao Xin Zhou Biotechnology company. Human undifferentiated gastric cancer cell line HGC-27 was donated by Harbin Medical University and well differentiation gastric cancer cell line

AGS was donated from East China Normal University. Notably, all cells were cultured in RPMI-1640 culture medium (Biological Industries; Kibbutz Beit-Haemek, Israel) containing 10% fetal bovine serum (Biological Industries; Kibbutz Beit-Haemek, Israel) and 1% penicillin-streptomycin (Solarbio; Beijing, China) at 37°C in a 5% CO2 incubator.

### 2.3. Cell transfection

MiR-34c-5p mimic, mimics control, miR-34c-5p inhibitor and inhibitors control were purchased from GenePharma (Shanghai, China). MAP2K1 overexpression plasmid, empty plasmid (Vector), and small interfering RNA (siRNA) targeting MAP2K1, and its negative control were obtained from Sangon Biotech (Shanghai, China). HGC-27 and MKN-28 cells were inoculated into 6-well cell culture plates at a concentration of 2×10^5^ cells per well. When cell growth density reached more than 80% transfection was performed using the Lipofectamine 3000 reagent (Invitrogen, Carlsbad, CA, USA) according to the manufacturer's instructions. After successful transfection verified by RT-qPCR, subsequent experiments were performed.

### 2.4. RT-qPCR

The miRNAs from human GC tissues and cell lines were extracted by using the E.Z.N.A.® miRNA Kit, and the total RNA from transfected GC cells were isolated using TRizol reagent (Thermo Fisher), respectively, according to the instructions provided by the manufacturer. The RNA was then reverse transcribed to cDNA by the the PrimeScript RT Reagent Kit (TaKaRa, Kyoto, Japan), then diluted the cDNA add to the followed amplification and quantification by real-time PCR with TB Green® Premix Ex Taq™ II kit (TaKaRa, Kyoto, Japan). U6 and GAPDH were used as internal references for miR-34c-5p and MAP2K1, and the relative expression levels of miRNA and mRNA were calculated by the 2 ^-△△ct^ method. The primer sequences are as follows: miR-34c-5p forward 5'-GCC AGG CAG TGT AGT TAG CT-3' and reverse 5'-ATC CAG TGC AGG GTC CGA GG-3'; MAP2K1 forward 5'-ATG TTT GGG TGC CAG GTG GA-3' and reverse 5'-GGT CGG CTG TCC ATT CCG TA-3'; U6 forward 5'-CGC TTC GGC AGC ACA TAT AC-3' and reverse 5'-TTC ACG AAT TTG CGT GTC AT-3'; GAPDH forward 5'-GAC TCA TGA CCA CAG TCC ATG C-3' and reverse 5'-AGA GGC AGG GAT GAT GTT CTG-3'

### 2.5. Western Blotting

Total protein of GC tissues and cells was extracted using Whole Cell Lysis Assay (KeyGEN, Jiangsu, China) following the manufacturer's instructions. Thereafter, the protein concentration was measured by the BCA Protein Assay Kit (Solarbio, Beijing, China). First, equal amounts protein were separated by sodium dodecyl sulfate-polyacrylamide gel electrophoresis (SDS-PAGE) and transferred onto polyvinylidene difluoride (PVDF) membranes (Millipore, Billerica, MA, USA). Then, the membranes were blocked with 10% skim milk for 1 hour and incubated with the corresponding specific antibodies overnight at 4°C. Next, the membranes were incubated with horseradish peroxidase-conjugated secondary antibodies (1:5000; Cwbio, Beijing, China) at room temperature for 2 h. Last, the detection were performed by enhanced chemiluminescence (ECL) and the proteins were visualized with an Amersham Imager 600 instrument (GE Healthcare, Little Chalfont, UK). The antibodies used as follows: E-cadherin (1:300), N-cadherin (1:1000), Vimentin (1:1000), MAP2K1 (1:1000), GAPDH (1:1000).

### 2.6. Cell clone formation experiment

Cell proliferation capacity was assayed by clone formation assay. The cell suspensions containing 1000 cells were inoculated in 6-well culture plates and cultured in an incubator for 14 days, after fixing with 4% paraformaldehyde, the cells were stained with 0.1% crystal violet. Colony formation was then determined by counting the number of stained colonies.

### 2.7. Transwell migration and invasion assay

The ability of cells to migrate and invade was measured with transwell chambers (8 *μ*m pore size, Costar, Cambridge, MA, USA). Typically, the matrix gel Matrigell (BD Biosciences, USA) was pre-coated onto the transwell chambers for measuring cell invasiveness, but in migration assays Matrigel is not used. Cells were resuspended in serum-free medium and adjusted to 5×10^5^ cells/ml, then inoculated into the upper chamber of the transwell, the lower chamber was supplemented with RPMI-1640 medium containing 10% FBS，and then the cells were cultured at 37°C and 5% CO_2_ for 24h. Transwell chambers were removed, and the cells in the upper chamber were gently wiped away using cotton swabs, the migrated and invaded cells were fixed in 4% paraformaldehyde for 20 min, 0.1% crystal violet was stained for 15 min, and finally, the number of cells penetrating the membrane was observed under the microscope and photographed.

### 2.8. Dual-luciferase reporter gene assay

The targeted relationship between miR-34c-5p and the 3'-UTR of MAP2K1 was verified by a luciferase reporter gene assay. First, the wild type (WT) fragment of MAP2K1 3'-UTR containing the predicted miR-34c-5p binding sites was inserted into the pmirGLO vector by Genecreate (Wuhan, China) to construct MAP2K1-WT. Then, the target-binding sites between MAP2K1 and miR-34c-5p were mutated, and the mutant type (MUT) fragment was cloned into the pmirGLO vector to construct MAP2K1-MUT. After verification by DNA sequencing, miR-34c-5p mimics or mimics control and MAP2K1-MUT or MAP2K1-WT were co-transfected into cells using Lipofectamine 3000. After 48 hours, transfected cells were collected and analyzed by the dual-luciferase reporter gene detection system (Promega, Madison, WI, USA) as protocol.

### 2.9. Statistical analysis

Data were statistically analyzed and graphed using SPSS 23.0 and Graphpad prism 8.0. Data were presented as the mean ± standard deviation, the significance of the difference was evaluated either by Student's t test or by one-way ANOVA, which was considered statistically significant when *P* < 0.05.

## 3. Results

### 3.1. MiR-34c-5p is downregulated in GC and its relation with poor prognosis

**Figure 1:**
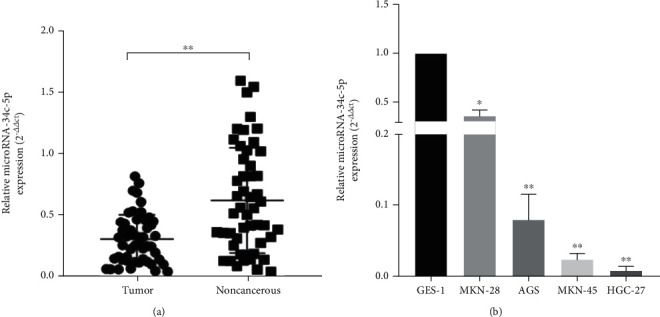
MiR-34c-5p is downregulated in GC tissues and cells. (A) The expression of miR-34c-5p in 50 pairs of GC tissues and adjacent paracancerous tissues. (B) The expression of miR-34c-5p in normal gastric mucosa epithelial cell line (GES-1) and four gastric cancer cell lines (AGS, MKN-28, MKN-45, HGC-27). ∗*P* < 0.05, ∗∗*P* < 0.01.

To study the role of miR-34c-5p in GC, we first analyzed miR-34c-5p expression in GC tissues and cells by using RT-qPCR. The results showed that miR-34c-5p was significantly downregulated in gastric cancer compared with adjacent control tissues (Figure 1(a)). As shown in Table 1, the expression of miR-34c-5p in GC tissues was closely related to the age, gender, and clinical stage of GC patients. In the group younger than 60 years old, low miR-34c-5p expression predominated; compared with female patients, male patients had higher miR-34c-5p expression levels, and the results showed that lower miR-34c-5p expression levels were associated with higher clinical stage, and the results were statistically significant. Moreover, the expression of miR-34c-5p in four gastric cancer cell lines (AGS, MKN-28, MKN-45, and HGC-27) and normal gastric mucosa epithelial cell line (GES-1) were next evaluated. It was found that the expression level of miR-34c-5p was also significantly lower in GC cell lines than in GES-1 cells (Figure 1(b)).

### 3.2. MiR-34c-5p inhibits proliferation, invasion, and migration of gastric cancer cells inhibits the malignant biological behaviors of GC cells

**Figure 2:**
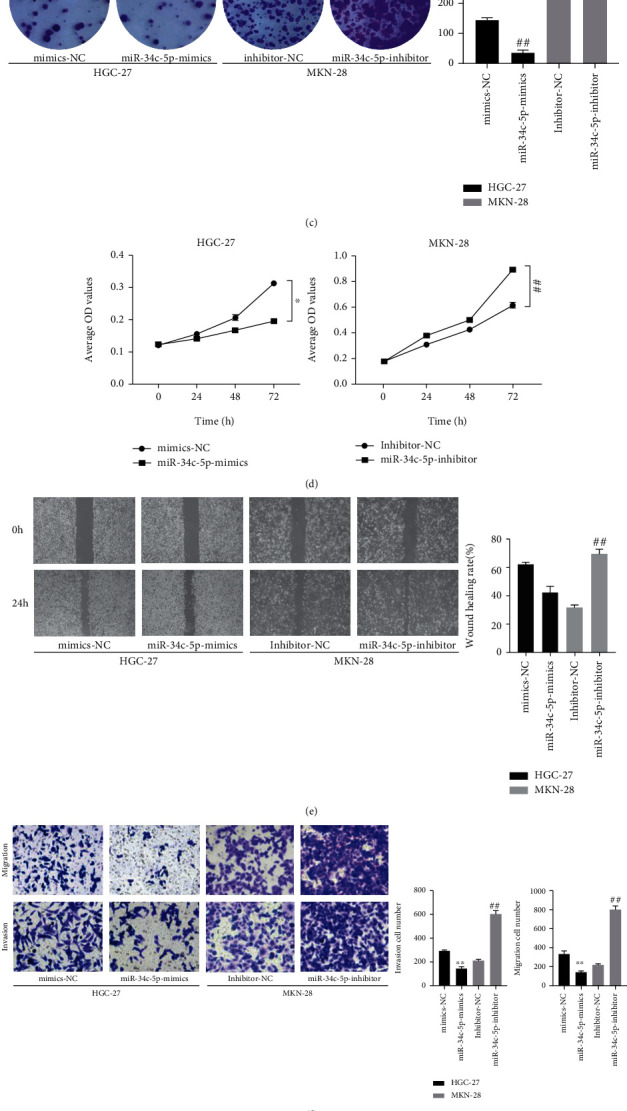
miR-34c-5p inhibits the proliferation, migration, and invasion of GC cells. (A, B) miR-34c-5p expression in HGC-27or MKN-28 cells transfected with miR-34c-5p mimic, mimics-NC or miR-34c-5p inhibitors, or inhibitor-NC were detected by qRT-PCR. (C) Proliferation ability of HGC-27 and MKN-28 cells was assessed by using colony formation. (D) CCK-8 assay detects the effect of cell proliferation viability after miR-34c-5p overexpression or knockdown.(E) The migration ability of cells was evaluated by wound healing assay. (F) The migration and invasion ability of cells were evaluated by transwell assay. (G) Western blot detection of protein levels of E-cadherin, N-cadherin, Vimentin, PCNA after miR-34c-5p overexpression or knockdown. ∗*P*< 0.05, ∗∗*P*< 0.01, ∗∗*P* < 0.01.

In order to assess the biological role of miR-34c-5p in GC, the effects of miR-34c-5p on cell proliferation, invasion, and migration ability were examined by clone formation assay, wound healing and Transwell, respectively. The miR-34c-5p mimic and inhibitor were transfected into HGC-27 and MKN-28 cells with relatively low and high expression of miR-34c-5p, respectively, to overexpress and knock down the expression of miR-34c-5p. It was subsequently verified by RT-qPCR that compared with the negative control, transfection of the miR-34c-5p mimic resulted in upregulation of miR-34c-5p expression in HGC-27, and transfection of the miR-34c-5p inhibitor led to downregulation of miR-34c-5p expression in MKN-28 (Figure 2(a) and 2(b)).

The results of the clone formation assay showed that transfection with miR-34c-5p mimic suppressed the colony forming ability of cells and conversely miR-34c-5p inhibitors promoted the colony formation (Figure 2(c)). CCK-8 assay of cell proliferation viability revealed that cell proliferation ability was significantly decreased after overexpression of miRNA-34c-5p, and in contrast knockdown resulted in a markedly enhanced proliferation ability of GC cells (Figure 2(d)). In addition, by wound healing assay, it was found that the lateral migration ability of GC cells which transfected with miR-34c-5p mimics was significantly inhibited, but the opposite result was found for transfection with miR-34c-5p inhibitors (Figure 2(e)). Moreover, Transwell assay showed that the invasion and migration ability of GC cells were significantly inhibited after miR-34c-5p overexpression, while knockdown of miR-34c-5p promoted the invasion and migration ability (Figure 2(f)). Finally, the expression of proliferation and invasion migration-related proteins was examined by western blot, which indicated that after miR-34c-5p overexpression, the E-cadherin expression was significantly increased, and N-cadherin, Vimentin, and PCNA expression were significantly decreased; and knocking down miR-34c-5p worked oppositely (Figure 2(g)). These results indicated that miR-34c-5p could inhibit the proliferation, migration, and invasion of GC cells, so as to inhibit the malignant biological behavior of GC.

### 3.3. MAP2K1 is the target gene of miR-34c-5p

**Figure 3:**
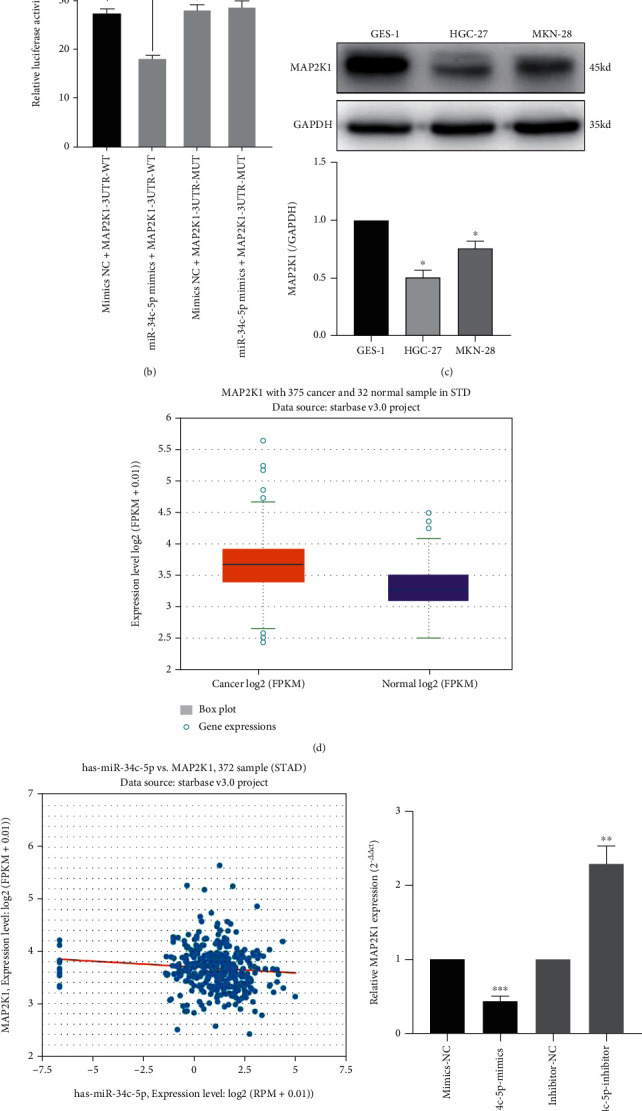
MAP2K1 is the target of miR-34c-p. (A) The predicted binding sequence between MAP2K1 3'-UTR and miR-34c-5p. (B) Dual-luciferase reporter gene assay was used to verify the binding relationship between miR-34c-p and MAP2K1 3'-UTR. (C) Western blot was performed to detect the protein level of MAP2K1 in normal gastric mucosal cells GES-1 and GC cells HGC-27 and MKN-28. (D, E) Starbase database was used to analyze the expression of MAP2K1 in GC tissues and its correlation with miR-34c-5p. (F) The expression of MAP2K1 after overexpression or knockdown of miR-34c-5p. ∗∗*P* < 0.01. ∗∗∗*P*< 0.001, ns: no significance.

To elucidate the potential role of miR-34c-5p in GC progression, we predicted the potential targets of miR-34c-5p by using TargetScan Database and selected MAP2K1 for further analysis. As shown in Figure 3(a), the 3'-UTR of MAP2K1 contains the complementary site of miR-34c-5p (Figure 3(a)). The binding of miR-34c-5p with MAP2K1 was confirmed by dual-luciferase reporter assay (Figure 3(b)). Overexpression of miR-34c-5p could demonstrably reduce the luciferase activity of MAP2K1-WT in 293 T cells, while it had no effect on the MAP2K1-MUT. We then examined the protein level of MAP2K1 in GC cells and found that the expression level of MAP2K1 protein in GC cells was significantly higher than that in GES-1 cells (Figure 3(c)). Moreover, miR-34c-5p was also negatively regulated with MAP2K1 at the RNA level. The expression level of MAP2K1 was significantly reduced after overexpression of miR-34c-5p, and conversely, MAP2K1 expression level was increased after knockdown of miR-34c-5p (Figure 3(f)). The expression levels of MAP2K1 in GC tissues and normal tissues adjacent to cancer were retrieved by starbase database, and the outcomes revealed that MAP2K1 mRNA were significantly higher in GC tissues than in paracancerous tissues (Figure 3(d)). In addition, the expression level of miR-34c-5p was negatively correlated with MAP2K1 expression in GC tissues (Figure 3(e)). In conclusion, the above data suggest that MAP2K1 is a direct target of miR-34c-5p in GC and it may play a vital role in GC progression.

### 3.4. MAP2K1 attenuates the inhibitory effect of miR-34c-5p in GC

**Figure 4:**
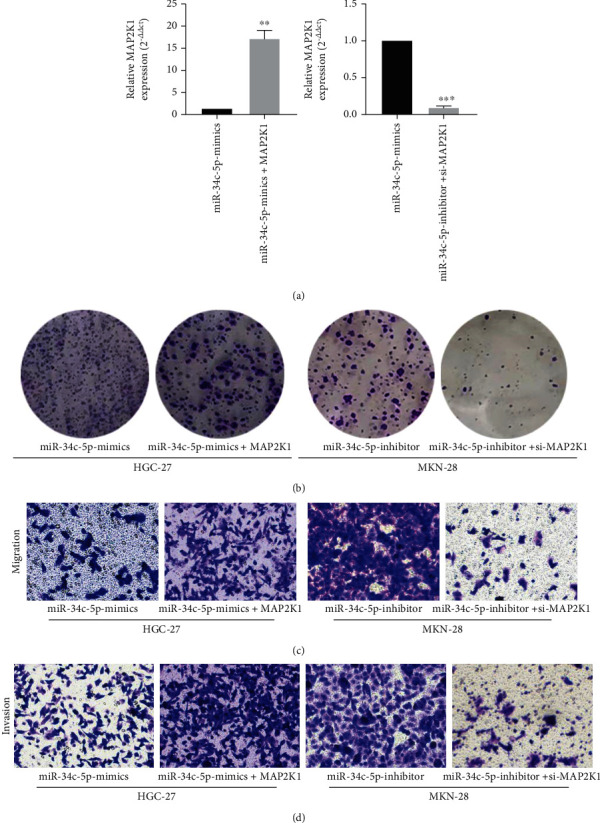
Overexpression of MAP2K1 attenuated the inhibitory effect of miR-34c-5p on GC cells. (A) RT-qPCR was used to detect MAP2K1 expression after co-transfection of PCMV-MAP2K1 with miR-34c-5p mimics or MAP2K1 siRNA with miR-34c-5p inhibitors. (B) Proliferation ability of GC cells was assessed after cotransfection of PCMV-MAP2K1 with miR-34c-5p mimics or MAP2K1 siRNA with miR-34c-5p inhibitor using colony formation. (C, D) The migration and invasion ability of cells were evaluated by transwell assay after cotransfection of PCMV-MAP2K1 with miR-34c-5p mimics or MAP2K1 siRNA with miR-34c-5p.

We verified if MAP2K1 expression can be targeted and inhibited by miR-34c-5p in GC via rescue experiments. Firstly, the overexpression plasmid PCMV-MAP2K1 was cotransfected into HGC-27 cells with miR-34c-5p mimic, and then MAP2K1 siRNA was cotransfected into MKN-28 cells with miR-34c-5p inhibitor. After that, the MAP2K1 expression was detected by RT-qPCR. The findings revealed that the expression of MAP2K1 was significantly higher in GC cells transfected with contemporaneous PCMV-MAP2K1 compared with miR-34c-5p mimic, and decreased significantly in GC cells transfected with MAP2K1 siRNA compared with miR-34c-5p finhibitor group (Figure 4(a)). Furthermore, MAP2K1 overexpression reversed the inhibitory effects of miR-34c-5p mimic on GC cell proliferation, invasion, and migration, and conversely, MAP2K1 interference reversed the promotive effects of miR-34c-5p inhibitor on GC cell proliferation, invasion and migration (Figure 4(b)–4(d)). The above results suggest that miR-34c-5p can perform the inhibitory effect on GC through its target MAP2K1.

## 4. Discussion

GC is a common gastrointestinal tumor worldwide [11], although there are few patients improved by surgical treatment, radiotherapy, and immunotherapy every year, the prognosis and 5-year survival rate of GC are still very poor [12, 13], and the main reason for the high mortality rate and poor prognosis of GC is the low early diagnosis rate, so it is of great importance to explore the early diagnostic markers of GC for the treatment of patients [14–16]. According to several studies, miRNAs are involved in the pathogenesis and development of GC, furthermore, an increasing number of miRNAs have been shown to be used as markers for diagnosis in GC treatment, and the dysregulated expression of miRNAs can remarkably affect prognosis [17, 18]. For instance, miRNA-204-5p exerts antitumor effects by inhibiting GC cell proliferation, invasion and migration, and promoting apoptosis, suggesting that miRNA-204-5p could be a potential target for the treatment of GC [19]. Compared with other miRNAs, miRNA-381 is highly sensitive and specific in GC diagnosis. Moreover, downregulation of miRNA-381 positively correlates with lymph node metastasis and progression of GC, indicating that miRNA-381 can be used as an early diagnostic marker for GC [20]. It has been reported that miRNA-34c-5p is lowly expressed in various cancers including ovarian, colon, and thyroid cancers [21–23] and acts as a tumor suppressor to participate in malignant biological behavior. Based on our previous study, we found that miRNA-34c-5p was also lowly expressed in GC tissues and cell lines, so it was hypothesized that miRNA-34c-5p could act as an oncogenic factor to regulate the malignant behavior of GC However, the function and mechanism of miRNA-34c-5p in GC have not been reported so far. Therefore, exploring the role of miRNA-34c-5p in the development of GC may lead to new markers for the diagnosis and treatment of GC.

To evaluate the effect of miRNA-34c-5p on the biological function of GC cells and its mechanism, firstly, we overexpressed or silenced miRNA-34c-5p in GC cells. Via a series of experiments, we found that overexpression of miRNA-34c-5p significantly inhibited the proliferation, invasion and migration of GC cells, but when miRNA-34c-5p was inhibited, the effect was reversed. Since the key to invasive migration of cancer cells is the occurrence of Epithelial-Mesenchymal Transition (EMT), and the markers of EMT occurrence include the reduction of the epithelial marker E-cadherin, and the upregulation of N-cadherin and Vimentin [24, 25]. Therefore, we examined the expression of these key proteins by western blot and found that overexpression of miRNA-34c-5p was able to significantly reduce PCNA, N-cadherin, and vimentin expression and markedly increase E-cadherin protein; inhibition of miRNA-34c- 5p had the opposite effect. As confirmed by our study, consistent with expectations, miRNA-34c-5p, which is lowly expressed in GC, has the ability to inhibit the proliferation, invasion and migration of GC cells, and functions as an oncogenic factor in GC.

Since miRNAs can inhibit mRNA translation by complementary pairing with the 3'-UTR, thus regulating target gene expression [26, 27]. For example, miRNA-30a-3p was found to inhibit GC cell proliferation and migration by targeting APBB2 [28]; miRNA-539 inhibited osteosarcoma cell proliferation, apoptosis, invasion and migration by targeting TRIAP1 [29]. MiRNA-34c-5p is a member of the miRNA-34 family, miRNA-34 family can inhibit tumor progression by promoting apoptosis and blocking the cell cycle [30]. And miRNA-34c-5p has been shown to exert oncogenic effects in renal cell carcinoma, oral squamous cell carcinoma and other tumors by targeting MMP2 and TRIM29 to inhibit cell proliferation, invasive and migration [31, 32]. Thus, finding the target genes of miRNA-34c-5p in GC could help us to investigate the mechanism by which miRNA-34c-5p inhibits GC progression. MAP2K1 is an important member of the mitogen-activated protein kinase family, also known as MEK1, which plays a role in cell proliferation and apoptosis. Cui et al. found that overexpression of miR-539 inhibited cell proliferation, migration, invasion and promoted apoptosis, thus inhibiting the progression of hepatocellular carcinoma, and this oncogenic function of miR-539 was accomplished by directly targeting and regulating MAP2K1 [33], Zhou et al. found that MAP2K1 is also a target for miR-149-5p, and miR-149-5p can regulate the proliferation, migration and invasion of hepatocellular carcinoma cells by targeting MAP2K1 [34]. It has also been shown that miRNA-101 affects the ERK/MAPK pathway by targeting MAP2K1 to regulate proliferation and apoptosis in diffuse large B-cell lymphoma [35]. In our study, MAP2K1 was identified as a potential target of miRNA-34c-5p through TargetScan database, and further bioinformatics analysis revealed that miRNA-34c-5p has a complementary binding site to the 3'UTR of MAP2K1, which was later confirmed by a dual luciferase reporter assay. Finally, MAP2K1 was found to be highly expressed in GC tissues by starbase database analysis, and the expression of miRNA-34c-5p was negatively correlated with MAP2K1. We further designed rescue experiments demonstrating that the miRNA-34c-5p mediated inhibition of GC cell proliferation, invasion and migration capacity was reversed by co-transfection with MAP2K1. Proved that miRNA-34c-5p played an oncogenic role by targeting binding to MAP2K1.

## 5. Conclusion

In summary, miRNA-34c-5p was lowly expressed in GC tissues and cells, and miRNA-34c-5p inhibited GC cell proliferation, migration and invasion by targeting MAP2K1. Our work elucidates the important role of the miRNA-34c-5p/MAP2K1 axis in the progression of GC, which provides possible biomarkers and targets for GC.

## Figures and Tables

**Table 1:** Correlation between miR-34c-5p expression and clinicopathologic characteristics of GC patients.

Characteristics	All cases (n=50)	miR-34c-5p expression		P value
		High(n=21)	Low(n=29)	
Gender				
Male	37	19	18	
Female	13	2	11	0.024
Age (years)				
>60	21	13	8	
≤60	29	8	21	0.015
Tumer size (cm)				
≥4	25	10	15	
<4	25	11	14	0.164
TNM stage				
I–II	19	12	7	
III–IV	31	9	22	0.017
Differentiation grade				
Poorly	28	10	18	
Well/moderate	22	11	11	0.320
T stage				
T1-2	18	5	13	
T3-4	32	16	16	0.132
N stage				
N1-N3	29	12	17	
N0	21	9	12	0.919

## Data Availability

The data used to support the findings of this study are included within the article.
